# Supplementary material for: Co-Delivery of a High Dose of Amphotericin B and Itraconazole by Means of a Dry Powder Inhaler Formulation for the Treatment of Severe Fungal Pulmonary Infections
Source: Pharmaceutics. 2023 Nov 8;15(11):2601. doi: 10.3390/pharmaceutics15112601 (PMC10675812; doi:10.3390/pharmaceutics15112601)
Supplement: Supplementary file 1 [file pharmaceutics-15-02601-s001.zip › pharmaceutics-2645085-supplementary.pdf]

Supplementary Materials: Co-Delivery of a High Dose of Amphotericin B and Itraconazole by Means of a Dry Powder Inhaler Formulation for the Treatment of Severe Fungal Pulmonary Infections

Salomé S. Celi, Raquel Fernández-García, Andreina I. Afonso-Urich, M. Paloma Ballesteros, Anne Marie Healy and Dolores R. Serrano

Results section – Box Behnken model

1. Yield

Summary (detailed tables shown below)

Source	Sequential p-value	Lack of Fit p-value	Adjusted R-Squared	Predicted R-Squared	
Linear	0,2474	0,0376	0,0945	-0,4147	
2FI	0,0546	0,0761	0,4321	-0,3021	Suggested
Quadratic	0,2114	0,0872	0,5572	-1,4710	
Cubic	0,0872		0,8259		Aliased

Source	Sum of Squares	df	Mean Square	F Value	p-value Prob > F	
Model	895,06	6	149,18	3,03	0,0590	
A-Antibi[illegible]os total	*253,58*	*1*	*253,58*	*5,15*	*0,0466*	
B-Flow rate	*81,79*	*1*	*81,79*	*1,66*	*0,2265*	
C-Leucina	*31,28*	*1*	*31,28*	*0,64*	*0,4440*	
AB	*175,30*	*1*	*175,30*	*3,56*	*0,0886*	
AC	*297,91*	*1*	*297,91*	*6,05*	*0,0337*	
BC	*55,20*	*1*	*55,20*	*1,12*	*0,3146*	
Residual	492,49	10	49,25			
Lack of Fit	*432,10*	*6*	*72,02*	*4,77*	*0,0761*	*t*
Pure Error	*60,39*	*4*	*15,10*			
Cor Total	1387,55	16				

2. Particle size

Summary (detailed tables shown below)

Source	Sequential p-value	Lack of Fit p-value	Adjusted R-Squared	Predicted R-Squared	
Linear	0,0199	0,6719	0,4083	0,1741	Suggested
2FI	0,8180	0,5377	0,2961	-0,5098	
Quadratic	0,8076	0,3348	0,1174	-2,5899	
Cubic	0,3348		0,2829		Aliased

Source	Sum of Squares	df	Mean Square	F Value	p-value Prob > F	
Model	3,05	3	1,02	4,68	0,0199	significant
A-Antibi[illegible]os total	*0,48*	*1*	*0,48*	*2,21*	*0,1612*	
B-Flow rate	*2,48*	*1*	*2,48*	*11,38*	*0,0050*	
C-Leucina	*0,099*	*1*	*0,099*	*0,46*	*0,5118*	
Residual	2,83	13	0,22			
Lack of Fit	*1,77*	*9*	*0,20*	*0,75*	*0,6719*	*not significant*
Pure Error	*1,05*	*4*	*0,26*			
Cor Total	5,88	16				

3. Aggregation state

Summary (detailed tables shown below)

**Source**	**Sequential p-value**	**Lack of Fit p-value**	**Adjusted R-Squared**	**Predicted R-Squared**	
Linear	0,0466	0,2288	0,3192	0,0130	Suggested
2FI	0,9958	0,1329	0,1203	-1,1143	
Quadratic	0,3704	0,1110	0,1754	-3,4440	
Cubic	0,1110		0,6320		Aliased

**Source**	**Sum of Squares**	**df**	**Mean Square**	**F Value**	**p-value Prob > F**	
Model	0,093	3	0,031	3,50	0,0466	significant
A-Antibi[illegible]os total	*0,092*	*1*	*0,092*	*10,45*	*0,0066*	
B-Flow rate	*2,645E-004*	*1*	*2,645E-004*	*0,030*	*0,8653*	
C-Leucina	*2,311E-004*	*1*	*2,311E-004*	*0,026*	*0,8740*	
Residual	0,11	13	8,830E-003			
Lack of Fit	*0,096*	*9*	*0,011*	*2,23*	*0,2288*	*not significant*
Pure Error	*0,019*	*4*	*4,773E-003*			
Cor Total	0,21	16				

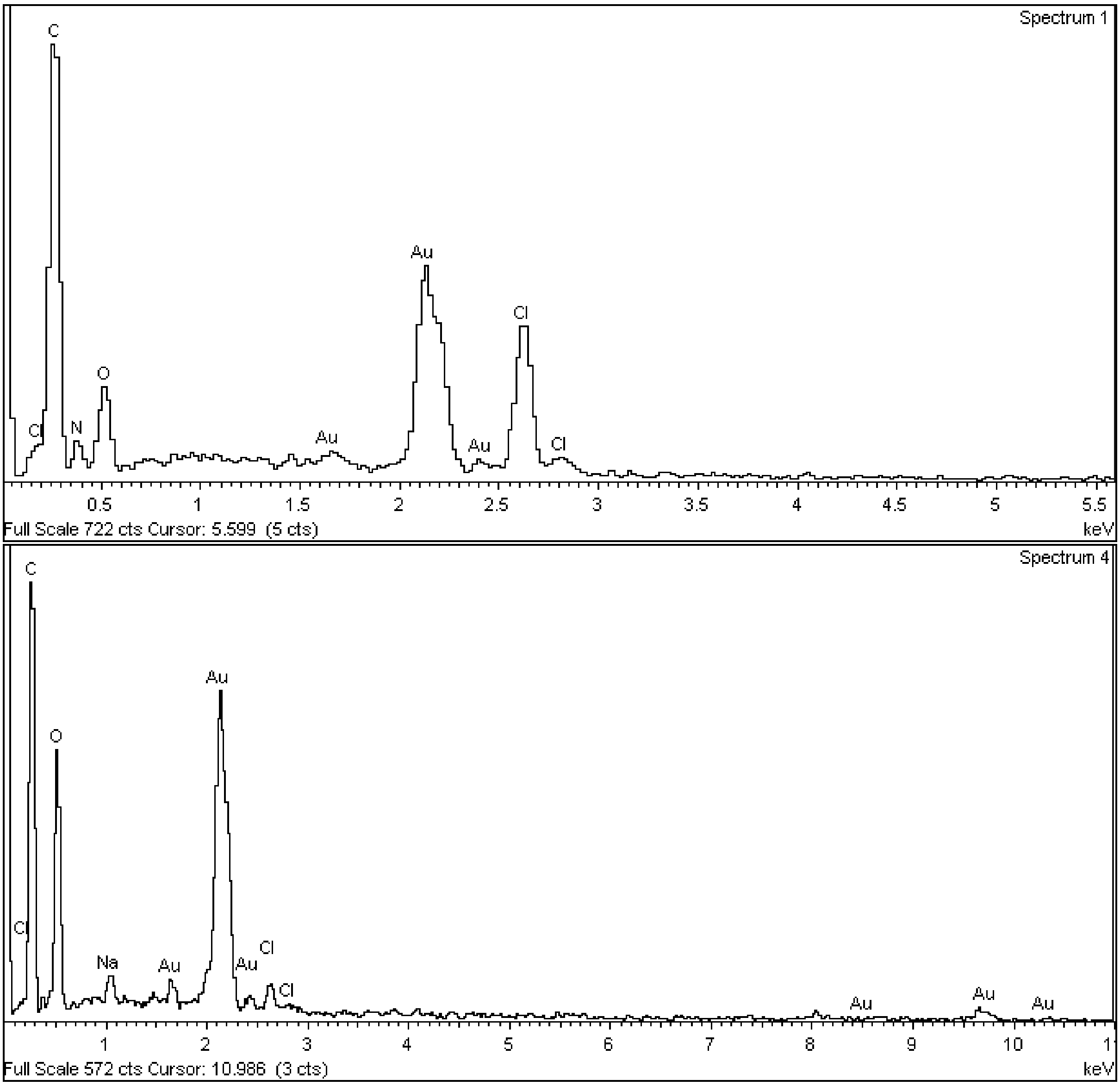

Figure S1. EDX spectra for F1.

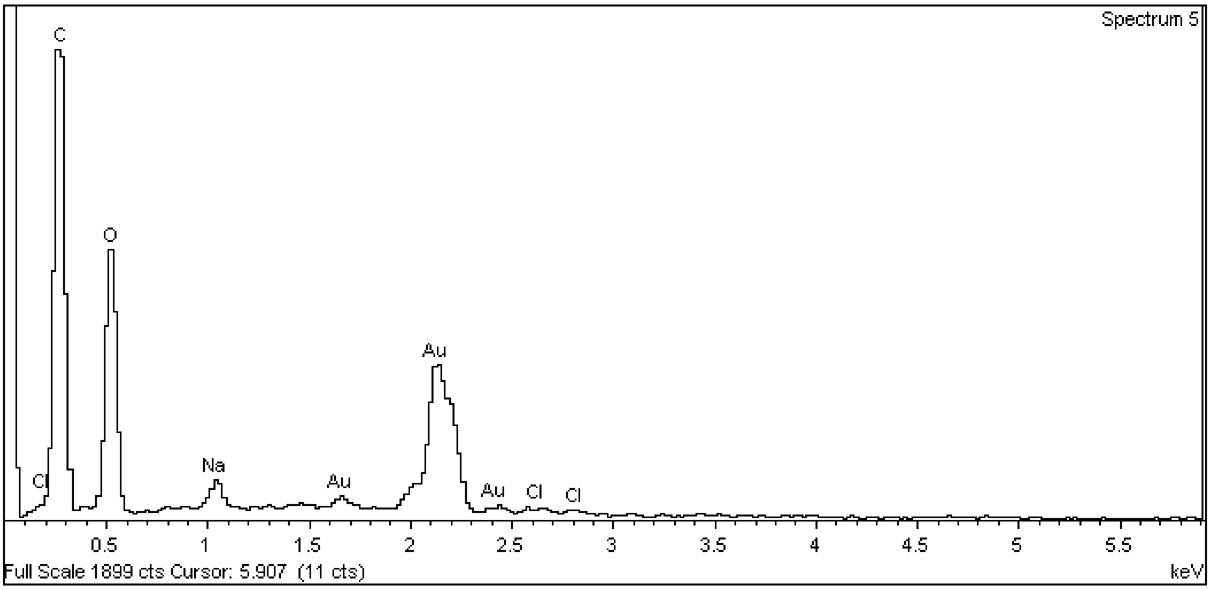

Figure S2. EDX spectra for F2.

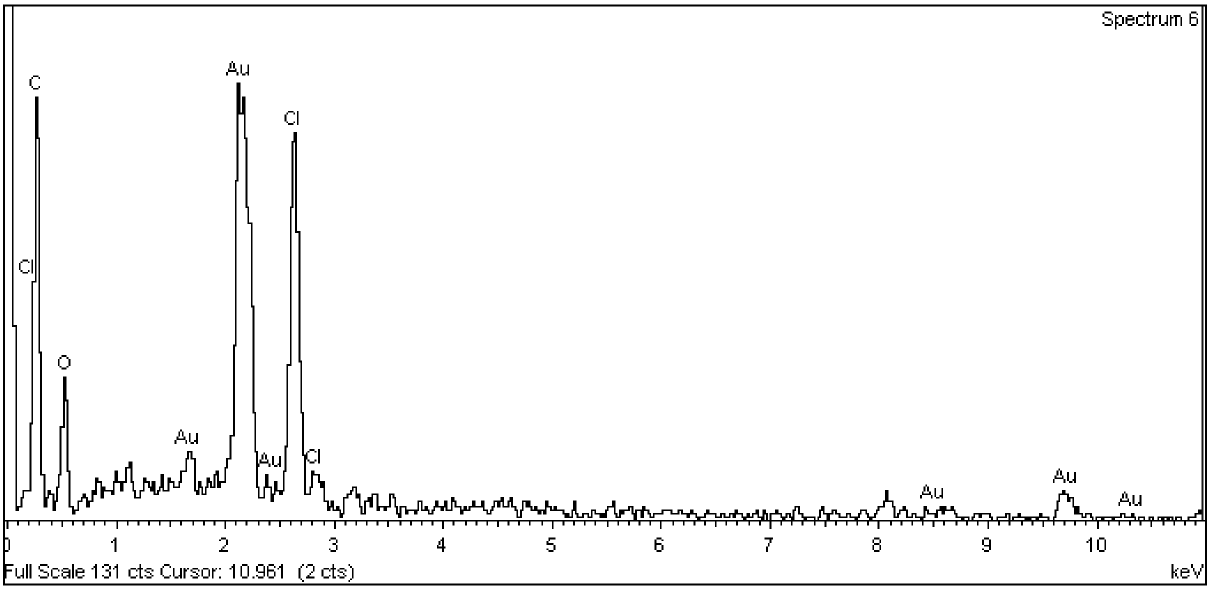

Figure S3. EDX spectra for F3
